# Exercise as a model to identify microRNAs linked to human cognition: a role for microRNA-409 and microRNA-501

**DOI:** 10.1038/s41398-021-01627-w

**Published:** 2021-10-08

**Authors:** Maria Goldberg, Md Rezaul Islam, Cemil Kerimoglu, Camille Lancelin, Verena Gisa, Susanne Burkhardt, Dennis M. Krüger, Till Marquardt, Berend Malchow, Andrea Schmitt, Peter Falkai, Farahnaz Sananbenesi, Andre Fischer

**Affiliations:** 1 German Center for Neurodegenerative Diseases, Department for Epigenetics and Systems Medicine in Neurodegenerative Diseases, Von Siebold Str 3A, 37075 Goettingen, Germany; 2 Developmental Neurobiology Laboratory, European Neuroscience Institute, Grisebachstrasse 5, 37077 Goettingen, Germany; 3 Interfaculty Chair for Neurobiological Research, RWTH Aachen University: Medical Faculty, Clinic for Neurology & Faculty for Mathematics, Computer and Natural Sciences, Institute for Biology 2, Worringer Weg 3, 52074 Aachen, Germany; 4 Department of Psychiatry and Psychotherapy, University Hospital, Ludwig-Maximilians-University Munich, Nußbaumstr. 7, 80336 München, Germany; 5 Department of Psychiatry and Psychotherapy, University Medical Center, Von-Siebold-Str. 5, 37075 Göttingen, Germany; 6 Laboratory of Neuroscience (LIM27), Institute of Psychiatry, University of Sao Paulo, 05403-010 São Paulo, Brazil; 7 German Center for Neurodegenerative Diseases, Research Group for Genome Dynamics in Brain Diseases, Von Siebold Str. 3A, 37075 Göttingen, Germany; 8 Cluster of Excellence “Multiscale Bioimaging: from Molecular Machines to Networks of Excitable Cells” (MBExC), University of Göttingen, Göttingen, Germany

**Keywords:** Epigenetics and behaviour, Molecular neuroscience

## Abstract

MicroRNAs have been linked to synaptic plasticity and memory function and are emerging as potential biomarkers and therapeutic targets for cognitive diseases. Most of these data stem from the analysis of model systems or postmortem tissue from patients which mainly represents an advanced stage of pathology. Due to the in-accessibility of human brain tissue upon experimental manipulation, it is still challenging to identify microRNAs relevant to human cognition, which is however a key step for future translational studies. Here, we employ exercise as an experimental model for memory enhancement in healthy humans with the aim to identify microRNAs linked to memory function. By analyzing the circulating smallRNAome we find a cluster of 18 microRNAs that are highly correlated to cognition. MicroRNA-409-5p and microRNA-501-3p were the most significantly regulated candidates. Functional analysis revealed that the two microRNAs are important for neuronal integrity, synaptic plasticity, and morphology. In conclusion, we provide a novel approach to identify microRNAs linked to human memory function.

## Introduction

MicroRNAs are 19-22 nucleotide long RNA molecules that regulate protein homeostasis via binding to a target mRNA thereby causing its degradation or inhibition of translation [1]. There is emerging evidence that microRNAs are essential for neuronal integrity and play a key role in cognitive function under physiological and pathological conditions [2–4]. Since microRNAs are extremely stable in cell-free environments and are resistant to freeze–thaw cycle they are investigated as biomarkers for various diseases, including brain diseases [5–7]. Interestingly, microRNAs can also act in a paracrine manner and participate in inter-organ communication [8–11]. This suggests that alterations of microRNA expression in the blood induced by environmental or disease-associated factors, reflect alterations in organs, the brain for example. Consequently, several studies analyzed the expression of microRNAs in blood and brain tissue of patients suffering from neurodegenerative and neuropsychiatric diseases. Interpretation of such data is; however, sometimes complicated because pathology accumulates often long before there are any clinical signs of cognitive decline so that patients with neurodegenerative or neuropsychiatric diseases are often only diagnosed at an already advanced stage of molecular pathology [12]. Moreover, follow-up experiments are rare leaving the field with a large number of candidate microRNAs which presently makes it difficult to decide on specific microRNAs for further translation studies [7, 13, 14]. Experimental approaches that would help to further refine the list of microRNAs that were shown to exhibit altered expression in patients or animal models for cognitive diseases are therefore of utmost importance. We reasoned that exercise could be a novel way to identify microRNAs linked to processes that control human cognitive function. Thus, it is well-established that exercise improves memory function in healthy humans as well as in mice which is accompanied by structural and functional changes in the brain [15, 16]. Moreover, a number of elegant studies suggest that blood-borne factors contribute to the beneficial effect of exercise on brain function and learning behavior [17, 18] leading us to the hypothesis that the analysis of circulating microRNAs upon exercise-induced cognitive enhancement would be a *bona fide* model system to identify microRNAs linked to human cognition (Fig. S1).

In this study, we analyzed the circulating microRNAome in blood samples of healthy human subjects that underwent a 3-month exercise protocol accompanied by cognitive phenotyping. We correlated the improvement of cognitive function to changes in the circulating microRNAome and were able to identify a microRNA cluster linked to cognitive enhancement. Two key microRNAs of this cluster, microRNA-409-5p and microRNA-501-3p, were also expressed in the human brain and were similarly upregulated in the hippocampus of exercising mice. Further mechanistic studies showed that these microRNAs are essential for neuronal plasticity. Both microRNAs are amongst the candidates that were reported to be decreased in patients suffering from neurodegenerative diseases suggesting that our experimental approach is suitable to improve the identification of microRNAs linked to cognitive function, thereby facilitating translation of basic research findings into clinical application.

## Materials and methods

### Human participants

Within the framework of a previously published clinical study, healthy volunteers were recruited between 2010 and 2013 in the Department of Psychiatry and Psychotherapy at the University Medical Center Goettingen [19]. Participants that did not have any previous medical history of mental disorder are analyzed in this study. The study was approved by the local ethics committee of the University Medical Center Goettingen and was in accordance with the declaration of Helsinki. All volunteers provided informed consent to use the data for research purposes. In this study, we included 19 of those individuals (14 men and 5 women) for high throughput sequencing as RNA isolation from blood samples failed for 2 individuals.

### Endurance training and blood collection

Healthy individuals participated in three months long endurance training, in which participants performed dynamic aerobic exercise three times a week for 30 min on bicycle ergometer machines (ergobike Premium 8, Daum electronic GmbH, Fuerth, Germany) and were subjected to computer-assisted cognitive remediation (CogPack) twice a week between week 6 and 12 [19] (for details see also [20]). The intensity of sessions was defined individually. Blood was collected before the start of the experiment and after the completion of the experimental protocol. Since the training was performed 3× week it is important to note that the final collection of blood was not performed directly after cycling to avoid immediate effects of exercise. Whole blood was drawn from the cubital vein and collected into PAXgene^®^ Blood RNA tubes (BD, Germany). Samples were incubated at room temperature for 2 h to ensure complete lysis of blood cells. The tubes were then transferred to −20 °C for 24–72 h and then transferred to −80 °C freezer for final storage until RNA isolation for microRNA expression analysis.

### Cognitive testing

Cognitive tests were performed before and after the aerobic training following by the protocol previously described Malchow et al. [19]. Processing speed and executive function were analyzed by Trail Making Test A (TMT-A) and B (TMT-B) (Reitan and Wolfson 1985). For measuring cognitive flexibility the Wisconsin card sorting test (WCST) 64 card version was used [21]. In order to test short-term memory (STM) and long-term memory (LTM) verbal learning memory test (VLMT) was performed. A list of 15 words is read 5 times to the participant. After each time, the number of words the participant can recall is recorded. An intrusion list of 15 different items is then presented and must be recalled. The number of words recalled after the first presentation of the original word list and the number of words recalled from the intrusion list is summed as an index of verbal STM. After the recall of the intrusion list, the participant is asked to recall the original list, immediately and after 30 min. These two measures are summed as an index of verbal LTM [22]. All raters and the persons performing the cognitive testing were blinded to the study interventions. The difference between the baseline at the beginning and after three months of training was analyzed.

### Animals

For primary neuronal culture experiments, CD-1 mice (Janvier Labs, France) were used. For exercise and behavior testing, 2-month-old male and female C57B6J (Janvier Labs, France) were used. Animals were housed under standard conditions maintaining an artificial 12 h light and 12 h dark cycle. Food and water were provided ad libitum. All animal experiments were performed according to the local guidelines and were approved by the local Animal Welfare Office of Goettingen University and the Lower Saxony State Office for Consumer Protection and food safety.

### Behavioral experiments in mice

C57Bl/6 mice were divided into sedentary and exercise groups (*n* = 10/10). Cages in both groups were identical and contained running wheels. In the sedentary group, the wheels were blocked so running was not possible. The total period of the experiment lasted 18 weeks. During that time the exercising group had permanent access to running wheels. The information from the wheels could be constantly monitored in order to later exclude potential non-running animals. Water maze experiments were performed as described previously [23]. Training protocol in the water maze paradigm usually ranges between 8 and 10 days of training. Since we expected to detect enhanced memory consolidation in the exercise group, animals were only subjected to 5 days of training before the probe test was performed 24 h later. While this protocol is usually not sufficient to establish robust memory in wild-type mice, it is sensitive to detect memory enhancement upon voluntary exercise [24].

### Collection of brain CA1 subregions

At the end of the exercise experiment, C57Bl/6 mice were sacrificed by cervical dislocation. Whole brains were isolated. Dissection of Cornu Ammonis 1 (CA1) region was performed under Leica MC170 HD stereo microscope using fine microsurgical instruments. The whole procedure was performed in ice-cold Dulbecco’s phosphate-buffered saline (DPBS, PAN-biotech GmbH, Germany) supplemented with ethylenediaminetetraacetic acid (EDTA)-free protease inhibitor cocktail (Roche Diagnostics GmbH, Germany). Fresh tissues were then snap-frozen in liquid nitrogen and stored at −80 °C.

### Mouse primary neuronal cultures

Pregnant CD-1 mice were euthanized by cervical dislocation. Pups at the embryonic stage 17 (E17) were quickly decapitated. Hippocampi were dissected using Leica MC170 HD microscope and pulled in a 15 ml tube filled with ice-cold DPBS (PAN-biotech GmbH, Germany). At the end of the dissection ice-cold DPBS has exchanged with pre-warmed DPBS and 2.5% trypsin–EDTA (Gibco, USA). In this solution, the samples were incubated in the water bath at 37 °C for 15 min. The reaction was then stopped by adding and mixing several times with warm processing media containing Neurobasal^®^ medium 1× (Gibco, USA), 10% fetal bovine serum (FBS, Gibco, USA) and 1% Penicillin–Streptomycin solution (Thermo Fisher Scientific Inc., USA). Next, the tissue was mechanically homogenized with the pipet and centrifuged in processing media for 5 min at 300*g*. The cell pellet was resuspended in maintenance media containing Neurobasal^®^ medium 1× (Gibco, USA), 2% B-27TM supplement (Gibco, USA), 1% Penicillin–Streptomycin solution (Thermo Fisher Scientific Inc, USA), and 1% GlutaMAX supplement (Gibco, USA) and filtered with 70 µm Falcon^TM^ cell strainer (Fisher Scientific, USA). Neubauer chamber was used to count cell numbers. The cell was then resuspended in an adjusted volume of maintenance media and seeded with density of 130,000 cells/well on 24-well plates. For the experiments including image acquisition, sterile glass coverslips with a diameter of 12 mm were placed before coating. The plates were prepared in advance by coating with 0.5 mg/L poly-d-lysine (PDL, Sigma-Aldrich, Germany), incubating at 37 °C for 1 h, and the following washing with distilled water three times. Totally, 30% of the volume was changed every third day. Cell cultures were treated at a day in vitro (DIV) ten or DIV seven for 48 h for RNA sequencing and imaging analysis, respectively.

### LNPs preparation

For microRNA manipulation in cell culture microRNA inhibitors, mimics, and scramble microRNA controls were purchased from Qiagen, USA. Next, they were encapsulated into manufactured lipid nanoparticles (LNPs) according to the manufacturer’s protocol (Neuro9 siRNA Spark, Precision NanoSystems, Canada). Lyophilized microRNA inhibitors, mimics, and controls were dissolved in a storage buffer provided by the company to a final concentration of 1 mM. Nucleic acids were mixed with formulation buffers so that the final concentration was 930 µg/mL. LNPs were formulated in manufacturer’s cartridges using NanoAssembler SparkTM device (Precision NanoSystems, Canada). Prepared LNPs were later stored at 4 °C. LNPs were given to each well in the concentration of 0.01 µg/mL followed by adding 10 ng of ApoE provided in the mentioned kit.

### Total RNA purification from human blood samples

Total RNA including small RNA was manually purified from whole blood samples using PAXgene^®^ Blood microRNA Kit (PreAnalytiX GmbH, Switzerland). Before the beginning of isolation, samples were put at room temperature for 2 h. The tubes were centrifuged at 4000*g* for 10 min and the supernatant was removed. After pellet resuspension in RNase-free, this step was repeated one more time. The pellet was then dissolved in a given BM1 buffer. Buffer BM2 and proteinase K were added and then the samples were incubated at 55 °C for 10 min. Samples were pipetted into lilac spin columns and centrifuged for 3 min at full speed. The supernatant was transferred into new tubes and isopropanol was added. Samples were pipetted again into new red spin columns and centrifuged at 10,000*g* for 1 min. The columns were then transferred into new processing tubes. Samples were washed once for 15 s with buffer BM3 and then incubated in DNase solution for 15 min at room temperature. Spin columns were washed again, and processing tubes were changed. Buffer BM4 was added and centrifuged at 10,000*g* for 2 min. Columns were dried by additional short centrifugation. Columns were placed into new tubes and buffer BM5 was applied onto the membrane. This step was repeated twice. After 1 min centrifugation at 10,000*g* samples were incubated at 65 °C for 5 min and immediately transferred on ice. Samples were then stored at −80 °C till further use.

### Total RNA purification from mouse brain samples and neuronal cultures

Total RNA extraction including microRNA was followed by adding Tri reagent (Tri Reagent, Sigma-Aldrich, Germany) and proceeding with RNA Clean & Concentrator^TM^-5 Kit (Zymo Research Europe GmbH). Totally, 800 µl Tri reagent was added per CA1 brain tissue and the tissues were homogenized using a mechanical homogenizer. After homogenization, 200 µl chloroform was added and the solution was mixed using a benchtop mixer. Samples were kept at room temperature for 3 min. Next, samples were centrifuged for 15 min at 12,000×*g* at 4 °C. This high-speed centrifugation resulted in the formation of two layers: aqueous phase on top and organic phase at the bottom. For RNA isolation, the aqueous phase was carefully isolated without disturbing the interphase and mixed with 1× volume of 100% Ethanol. The solution was then transferred to zymo spin columns provided by RNA Clean & Concentrator kit and centrifuged for 1 min at 12,000×*g*. Columns were washed with 400 µl RNA Wash Buffer followed by incubation in DNase digestion mix (DNase I + buffer) for 15 min at room temperature. After incubation, 400 µl RNA prep buffer was added to the column and centrifuged for 1 min at 12,000×*g*. The flow-through was discarded and the spin columns were washed two times with the RNA wash buffer. While the first washing step involved centrifugation for 1 min at 12,000×*g*, during the second wash, spin columns were centrifuged for 2 min at 12,000×*g*. To dissolve RNA, 22 µl of DNase/RNase-Free Water was carefully added to the membrane of the zymo spin column and RNA was collected in RNAase-free 1.5 ml Eppendorf tubes after centrifugation at 12,000×*g* for 1 min. The concentration of RNA was measured using Nanodrop^TM^ 2000 Spectrophotometer (Thermo Fisher Scientific Inc, USA). Isolated total RNA was stored at −80 °C until further use.

### Real-time quantitative PCR

First, complementary DNA (cDNA) was synthesized from the total RNA pool using miScript II RT Kit (Qiagen, USA) for quantitative polymerase chain reaction (qPCR). MiScript SYBR^®^ Green PCR Kit (Qiagen, USA) was used for microRNA detection. Quantification of mature microRNAs was done on LightCycler^®^ 480 Instrument II (Roche Diagnostics GmbH, Germany) according to the manufacturer’s recommended protocol. RNU6B was used as a control for the normalization of values. Relative expression was analyzed using 2^−ΔΔc(t)^ method as described before [25].

### Small RNA sequencing

NEBNext^®^ Small RNA Library Preparation Set (Neu England Biolabs, USA) was used to generate high quality microRNAome data from human blood samples. Briefly, for the generation of microRNAome data, the total amount of 100 ng of RNA was taken for further cDNA preparation, fragmentation, adapter ligation, and hybridization. After pooling the libraries together, polyacrylamide gel electrophoresis was run for size selection. The insert size of 150 base pairs (bp) was chosen for further quantification and purification. Sequencing of 2 nM concentration was performed on HiSeq 2000 sequencing system (Illumina, USA) using a 50 bp single read setup. Sequencing data were demultiplexed using CASAVA v1.8 (Illumina, USA) and raw sequencing files in the format of fastq were generated.

### Bioinformatic processing of microRNA sequencing data

Analysis of the data was performed as previously described [25]. The quality of the sequencing data was investigated using FastQC v0.11.15 software. The raw sequencing reads were mapped to the reference genome and microRNA counts were generated using miRDeep2 package following the developer’s instruction. hg38 (UCSC) was used (UCSC) as a reference genome.

### Co-expression analysis of microRNA sequencing data

Weighted gene co-expression network analysis was performed using the WGCNA R package (v.1.61). First, microRNA counts were normalized to library size followed by transformation into log2 values. A quality *z*-score was calculated for each sample, and samples with low quality (*Z* > 2.5 or *Z* < −2.5) were defined as outliers and removed from further analysis. Thus, one sample was filtered out of the analysis. Surrogate variables were determined by sva R package and the effects of the covariates were adjusted using a mixed linear model. Pair-wise bi-weighted mid-correlations between microRNAs were calculated and a threshold power of 13 was chosen based on approximate scale-free topology and used to calculate the pair-wise topological overlap between microRNAs in order to construct a signed network. Identification of the co-expressed modules was performed with the minimum module members of 10 and deepsplit size 3. Modules with modular eigengenes (MEs) being highly correlated were merged using mergeCloseModules function setting the dissimilarity correlation threshold at 0.15. Module membership score was set to 0.40 to further filter the microRNA members in a given module. Different modules were summarized as networks of MEs, which were then correlated with the composite cognitive score calculated as mentioned above.

Networks of MEs were then generated for different modules and then used to correlate with available phenotypic information. Moreover, MEs were used to compare the expression of modules between time points using nonparametric tests. Moreover, MEs were correlated with the results from cognitive tests performed before and after exercise.

### mRNA sequencing

TrueSeq^®^ Library preparation kit (Illumina, USA) was used as described before [25]. RNA quality was checked on 2100 Bioanalyzer Instrument (Agilent Technologies, USA) and 500 ng of RNA was used as input for library preparation. Libraries were quantified using a Qubit 2.0 Fluorometer (Life Technologies, USA). The concentration of 2 nM was used for 50 bp single-end sequencing run on a HiSeq 2000 sequencing platform (Illumina, USA). Base files were translated to fastq files by bcl2fastq conversion software v.2.18.0. The quality of the files was checked using FastQC(v0.11.15). The reads were mapped to mm10 genome using STAR aligner v2.5.2b. Count files were generated using featureCounts software of subread package v1.5.1.

### Differential expression analysis of microRNA and mRNA

microRNAs with a reads count of at least five in one-third of all the samples were selected for differential expression analysis. Expression data were normalized to library size and log2 transformed. Unwanted surrogate variables were determined using sva. Adjustment of the normalized expression for unwanted effect due to biological covariates (e.g., age, gender, and education), and those surrogate variables were performed using a mixed linear model as described before [25]. Limma R package was used to test for differential expression. De-regulated microRNAs with adjusted *p* value < 0.05 were considered as significantly changed.

### Gene ontology analysis

To perform gene ontology analysis for 18 microRNAs in midnightblue cluster, confirmed target genes were retrieved from miRTarBase database (version 8) (http://mirtarbase.cuhk.edu.cn/php/index.php). Gene ontology (GO) analysis on those target genes was performed using an online tool (http://geneontology.org/) and statistically significant GO terms were retrieved (FDR < 0.05, Benjamin–Hochberg corrected). The significant GO terms were further filtered based on the previous curation of GO annotation relevant for dementia research (from Alzheimer’s Disease Association at UCL and Alzheimer’s project at the University of Toronto) and similar GO terms were merged into clusters using GO semantic similarity. The parental GO term was selected for further analysis. A cumulative *p* value of the parental GO term was calculated using Fisher’s combined probability test and was adjusted with the Bonferroni correction. Parental GO terms with adjusted *p* value < 0.05 were considered as significant.

For GO analysis of the deregulated mRNAs from manipulation of hippocampal neurons, analysis was performed using an online tool (http://geneontology.org/), and statistically significant GO terms were retrieved (FDR < 0.05, Benjamin–Hochberg corrected). Analysis was performed for up- and downregulated genes separately.

### Cell viability and neurite outgrowth assay

Cell viability was investigated using ReadyProbes^®^ Cell Viability Imaging Kit (Cat R37609, Thermo Fisher Scientific). In brief, 2 drops/ml of NucBlue and NucGreen reagents were added to the cells. After 1 h of incubation, fluorescence signals for NucBlue (excitation/emission: 360/460 nm) and NucGreen (excitation/emission: 504/523) were measured using a fluorescence plate reader.

For neurite outgrowth assay, a neurite outgrowth staining kit (Cat. A15001, Thermo Fisher Scientific) was used. Briefly, 1× working solution of Cell Membrane Stain was prepared by diluting it 1000-fold in Dulbecco’s Phosphate-Buffered Saline containing calcium and magnesium (Cat. no. 14287-080). The neuronal medium was removed and cultures were washed once with pre-warmed PBS. Totally, 400 µl of 1× membrane stain solution was added per well of 24-well plate containing neuronal cells, and cells were incubated for 21 h at 37 °C in a dark environment. After incubation, membrane stain solution was removed and cells were washed once with pre-warmed PBS. 1× working solution of the Background Suppression was prepared by diluting it 100-fold into DPBS containing calcium and magnesium (Cat. no. 14287-080) and added to each well (400 µl/ well of 24-well plate). The fluorescence signal of the membrane stain [Excitation 555 (nm), emission 565 (nm)] was quantified using a fluorescence plate reader.

### Immunohistochemistry

After 72 h treatment with LNPs as described above cell cultures that were growing on glass, coverslips were fixed at DIV ten with 4 % Paraformaldehyde (Sigma-Aldrich, Germany) for 30 min on a shaker at room temperature. They were then quenched in a 100 mM NH4Cl solution (Merck, Germany). For synapses quantification, coverslips were then washed and permeabilized three times for 10 min and then blocked in 0.1% Triton-X (Merck, Germany), 3% bovine serum albumin (AppliChem GmbH, Germany) in PBS for 1 h. Primary antibodies were then applied in 1:500 dilution for 1 h. After washing three times with PBS (5 minutes/wash), secondary antibodies were added in 1:200 dilution and incubated for 1 h. Before mounting coverslips on glass slides using mowiol (Merck, Germany), they were again washed three times using PBS. The whole procedure was done on a shaking platform at room temperature. Guinea pig Synaptophysin 1 (SySy, Germany), rabbit postsynaptic density (PSD)-95 (Cell Signaling Technology, Germany), microtubule-associated protein 2 (MAP2) (SySy, Germany) were used as primary antibodies. Corresponding secondary antibodies were donkey-anti-guinea Cy3 (Jackson Imm., UK) and goat-anti-rabbit Abberrior STAR 635p (Abberrior Instruments GmbH, Germany). For spine density analysis the coverslips were quickly washed with PBS three times. Dioctadecyl-3,3,3′,3′-Tetramethylindocarbocyanine Perchlorate (DiI) Stain (InvitrogenTM, US) dye was then applied. Five to six crystals were added on top of the coverslips and 200 µl of PBS was added. Total incubation time was 10 min on a benchtop shaker at room temperature. Afterward, the coverslips were washed with PBS till all the crystals were not visible anymore. The plates with 1 mL of PBS were placed on a shaker overnight at 4 °C. The coverslips were then washed three times and mounted using Fluoromount-G^™^ (Invitrogen, Germany).

### STED microscopy and image analysis

Stimulated emission depletion (STED) microscope Leica TCS SP5 (Abberrior Instruments GmbH, Germany) with the 100× oil-immersion objective and photomultiplier signal detector was used for acquiring high-resolution images of Synaptophysin 1, PSD-95, and MAP2 stained primary neuronal cultures. The areas of interest were defined randomly and the images were obtained in one plane. Analysis of colocalization of pre- and post-synaptic markers was performed using SynQuant plugin in ImageJ v. 2.0.0-rc-69/1.52p.

For DiI stained coverslips STEDYCON system installed on Leica microscope DMi8 with 63× oil-immersion objective was used. Default parameters for Cy3 wavelength were chosen. The whole stack images were obtained. Z-stack was defined automatically by STEDYCON smart control software. Stack images were later merged in ImageJ v.1.8.0_202. Spine density on a given dendritic length was defined manually using the NeuronJ plugin. The data were plotted and statistical analysis using parametric t-test was performed using Prism 8.3.1 program (GraphPad Software LLC, USA).

### Multi-electrode assay

E17 hippocampal regions were extracted as described above. After centrifuging with 300*g* for 5 min at room temperature cells were resuspended in NbActiv4^®^ neuronal culture medium (BrainBits, USA). Cells were counted, mixed with 1 µg/mL laminin (Merck, Germany), and plated at a density of 15,000 cells/µL into Lumos lens lid 48-well microelectrode array (MEA) plates equipped with 16 electrodes in each well. These wells were coated with 0.05 mg/L PDL in advance. 30 % of media change was conducted every third day. At DIV seven spontaneous basal activity was recorded. Afterward, cells were treated with LNPs containing micro-RNA inhibitors mix or scramble RNA as a control in the same concentration as described above. Starting from DIV ten spontaneous neuronal activity was recorded using the Maestro Apex Platform (Axion Biosystems, USA). Every 3 h during 24 h the measurements were recorded for 10 min. The data was later extracted and analyzed using neuronal module AxlS Navigator software (Axion Biosystems, USA). All the values were plotted and analyzed using the Prism 8.3.1 program (GraphPad Software LLC, USA).

### Statistical analyses

Statistical testing for sequencing analysis of sequencing experiments is described in the corresponding section and in the figure legend. All the statistical analyses mentioned in the main text have performed either prism (version 8) or in RStudio (v 1.4.1106).

## Results

### Circulating microRNAs linked to exercise-induced enhanced cognitive function

**Fig. 1:**
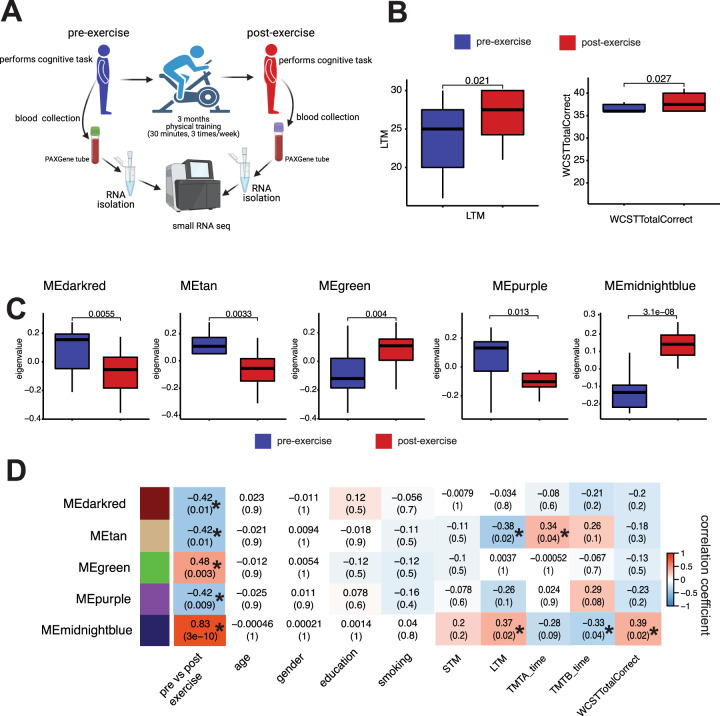
Circulating miroRNAs are correlated to exercise-mediated cognitive improvement. **A** Experimental scheme. Healthy volunteers (*n* = 19; 14 male and 5 females, 39 ± 10 years of age) participated in 3-month long physical exercise training. Cognitive performance was assessed using various psycho-cognitive tests prior (pre) and after (post) the experiment. Blood was collected at pre and post time points and RNA isolated from total blood was subjected to generate high-quality small RNAome profile. **B** Left panel: Long-term memory (LMT) measured via the VLMT and performance in the WCST (right panel) as measured by the number of correct choices (TotalCorrect) was significantly increased when comparing pre- vs. post-exercise. **C** Box plots showing the eigenvalue-expression of five microRNA co-expression modules (ME) that significantly differ when comparing pre- vs. post-exercise. **D** Heatmap showing a correlation between the eigenvalue-expression of five microRNA co-expression modules from (**C**) and the clinical traits. Each row represents a ME, each column corresponds to a trait. Each cell shows the corresponding correlation, *p* values are shown in brackets. The values are color-coded based on direction and degree of correlation. Blue represents negative correlation and red represents positive correlation. *p < 0.05 was considered significant. The horizontal line in the box plot represents the median, the box spans 25 and 75% quantile, and the whiskers represent the smallest and largest values in the 1.5× interquartile range.

To identify microRNAs that might play a role in synaptic plasticity and memory function we decided to analyze the circulating microRNAome in healthy individuals (*n* = 19; age: 39 ± 10 years) (Table S1) before and after a 3-month period of aerobic exercise, a procedure known to improve cognitive abilities [19]. Cognitive function was assessed before the first exercise session (pre-exercise) and blood was collected via Pax-gene tubes. Similarly, cognitive function was analyzed and blood was collected after 3 months of exercise (post-exercise) [19]. We then isolated RNA from the blood samples and performed smallRNA sequencing (Fig. 1A). Three-month of physical exercise had no significant effect on body weight, blood pressure, triglyceride, cholesterol, and glucose levels (Fig. S2). Neuropsychological testing to evaluate visual attention, mental flexibility, and memory was performed before the experiment and after the completion of the 3-month exercise. Speed of processing was analyzed via the trail-making tests (TMT) A and B, executive function was tested via the WCST while STM and LTM were evaluated by the VLMT. There was a nonsignificant trend for improved STM and the performance in the TMTs when comparing the pre- and post-exercise data (Fig. S2). LTM for the recall or words and cognitive flexibility measured by the total number of correct choices in the WCST was significantly improved after 3-month of exercise (Fig. 1B). Having confirmed that the employed exercise protocol affects cognitive function, we decided to analyze the smallRNA sequencing data. First, we performed a weighted co-expression analysis and identified 33 microRNA modules/clusters (Table S2). Next, we asked if the eigen-expression of any of these microRNA clusters would differ when comparing the pre- vs. the post-exercise datasets. Among 33 modules (Figs. S3), 5 modules exhibited a significantly different expression with the midnightblue cluster being the most significant (Fig. 1C, D). Altered expression of the five microRNA clusters upon exercise was not related to age, gender, education, or smoking habits (Fig. 1D). Next, we correlated the expression of the five microRNA clusters to the performance during the pre- and post-exercise neuropsychological testing. While the MEtan cluster was significantly correlated to performance in the LTM and TMT-A test, the most significant changes were observed for the midnightblue cluster (Fig. 1D). The midnightblue cluster was positively correlated to LTM performance in the VLMT and to the performance in WCST (Fig. 1D). It was also significantly correlated to the performance in TMT-B (*p* = 0.04) while a nonsignificant trend was observed in the TMT-A test (*p* value 0.09) (Fig. 1D). In summary, these data suggest that especially the expression of the midnightblue microRNA module is linked to better cognitive performance in response to exercise.

### Exercise-induced expression of microRNA-409-5p and 501-3p in humans and mice

**Fig. 2:**
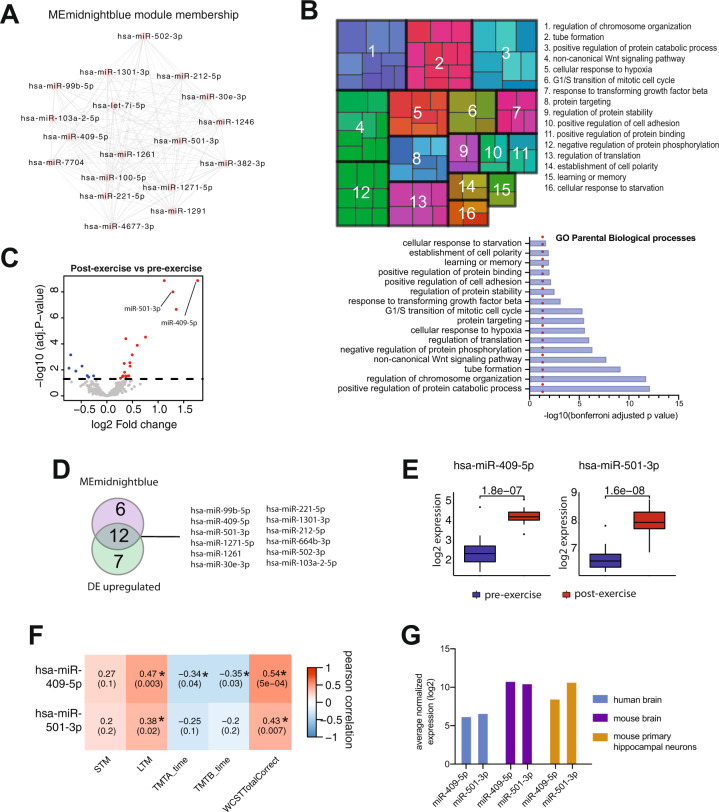
microRNA-409-5p and microRNA-501-3p are linked to exercise-mediated cognitive improvement. **A** MicroRNA network of MEmidnightblue representing the 18 microRNAs and their interconnectivity. **B** Gene ontology (GO) terms of biological processes regulated by deregulated microRNAs from MEmidnightblue. The analysis was limited to the confirmed microRNA target genes retrieved from miRTarBase database (version 8). Statistically significant GO enrichment terms for those genes were determined (FDR < 0.05, Benjamin–Hochberg corrected) based on the analysis performed using the gene ontology tool (http://geneontology.org/). For relevant GO terms related to brain functions, the significant GO terms were filtered based on the previous curation of GO annotation relevant for dementia research (from Alzheimer’s Disease Association at UCL and Alzheimer’s project at the University of Toronto). Similar GO terms were merged into clusters using GO semantic similarity and the parental GO term was highlighted for visualization. Small squares (thin width) within a large box (thick width) represent sister GO terms that belong to the given parental GO term. Numbers denote the unique parental biological processes. Barplot displays 16 parental GO terms that were statistically significant (*p* < 0.05, Fisher’s combined test, adjusted with Bonferroni test). The red dot line highlights the significance cut-off. (**C**). Volcano plot of differentially expressed microRNAs between samples collected before and after exercise. The *x*-axis shows fold changes in expression (log2), blue represents downregulation, red represents upregulation. The *y*-axis shows the adjusted *p* value (−log10) scale for the analyzed microRNAs. Values above the dashed line are considered significantly deregulated with adjusted *p* value < 0.05. Non-significant microRNAs are depicted in gray color. Each dot represents one single microRNA. **D** Venn diagram representing common genes between differentially regulated microRNAs when compared pre - vs. post-exercise samples and the microRNAs of MEmidnightblue module. **E** Expression of microRNA-409-5p and microRNA-501-3p when comparing pre- vs. post-exercise samples. The horizontal line in the box plot represents the median, the box spans 25 and 75% quantile, and the whiskers represent the smallest and largest values in the 1.5× interquartile range. *y*-Axis represents the normalized read counts. Two-tailed *t* test, unpaired. **F** Heatmap showing a correlation between the expression of microRNA-409-5p and microRNA-501-3p in human blood and the clinical traits. **G** Average expression of microRNA-409-5p and microRNA-501-3p in post-mortem human brain, healthy human blood, mouse hippocampus, and primary hippocampal neurons.

The midnightblue cluster consists of 18 microRNAs that are characterized by high intramodular connectivity (Fig. 2A). To provide first insight on the function of these microRNAs, we retrieved their experimentally confirmed target genes and performed an advanced gene-ontology analysis (see “Methods” for detail). Although these data have to be interpreted with care, the analysis revealed a number of biological processes linked to cognitive abilities, learning, or memory (Fig. 2B). To further identify the most relevant microRNAs within this cluster we decided to employ an alternative approach to the WGCNA analysis and performed a differential expression analysis comparing the microRNAome before and after exercise. Twenty-six microRNAs were differentially expressed (Fig. 2C, upregulated: 19; downregulated: 7) (Table S3). Notably, 12 of the significantly upregulated microRNAs also belong to the midnightblue cluster (Fig. 2D). Among the 12 microRNAs, microRNA-409-5p, and microRNA-501-3p were amongst the most significantly altered microRNAs (Fig. 2E). The expression levels of both microRNAs were also significantly correlated to LTM and the performance in the WCST (Fig. 2F). Expression changes of microRNA-409-5p were furthermore correlated to the performance in the trail-making tests (Fig. 2F). To provide further evidence that the expression changes observed in circulation might indicate an important role for microRNA-409-5p and microRNA-501-3p in memory function, we first asked if these microRNAs are also expressed in the brain. Both microRNAs were highly expressed in the post-mortem human brain of individuals that did not suffer from any mental disease, as well as in the mouse brain and in mouse hippocampal neurons (Fig. 2G).

**Fig. 3:**
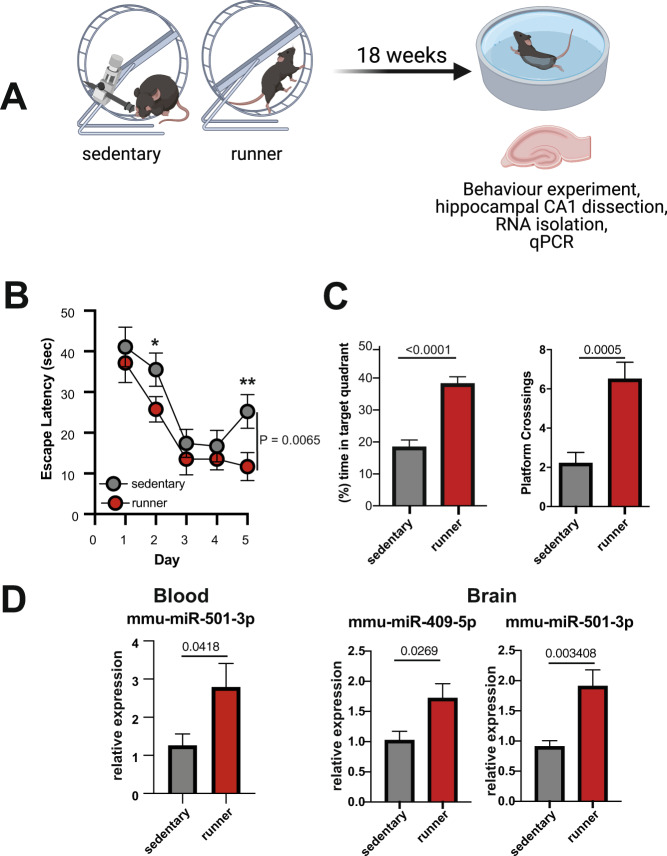
Increased expression of microRNA-409-5p and microRNA-501-3p in the hippocampus of mice upon exercise. **A** Experimental design. Mice of the experimental group (runners; *n* = 10) had free access to running wheels for voluntary exercise during a period of 18 weeks. Mice of the control group (*n* = 10) were housed under similar conditions but the running wheels were blocked (sedentary). After 18 weeks mice were subjected to a water maze-based spatial memory test and subsequently, hippocampal sub-regions were microdissected for molecular analysis. **B** Escape latency during the 5 days of training. Two-way ANOVA revealed a significant difference amongst groups (*P* = 0.0065, *F* = 7.7). Post hoc analysis revealed significant differences when comparing the performance on day 2 and day 5 amongst groups (^*^P < 0.1, unpaired *t* test; ^**^P < 0.05, unpaired *t* test). **C** Left panel: The time spent in the target quadrant during a probe test performed after 5 days of training was significantly increased (19.86 ± 3.14; mean ± SEM) in the runners-group (*n* = 10/group, unpaired *t* test, parametric, two-tailed). Right panel: Similarly, the number of platform crossings during the probe test was significantly higher in the runners-group (*n* = 10/group, unpaired *t* test, parametric, two-tailed). **D** Left panel: Bar graph depicting qPCR results showing increased expression of microRNA-501-3p in blood samples collected from mice before and after completion of the exercise procedure (*n* = 8/group unpaired *t* test, parametric, two-tailed). Please note that we could not detect microRNA-409-5p in blood samples from mice. Right panel: Bar graphs showing the qPCR expression of microRNA-409-5p and microRNA-501-3p in the hippocampal CA1 region. MicroRNA levels were increased in the runners-group (*n* = 9/group, unpaired *t* test, parametric, two-tailed). Error bars indicate mean ± SEM.

Since microRNA-409-5p and microRNA-501-3p were expressed in human blood and brain as well as in the mouse brain, we reasoned that the mouse would be a suitable model to test if exercise affects the expression of these microRNAs in the brain, a question that cannot be addressed easily in humans. Thus, mice were subjected to an established exercise protocol, and the hippocampal CA1 region, a brain region intimately involved in memory function in mice and humans, was isolated after the completion of the experiment (Fig. 3A). Mice housed under similar conditions but without exposure to exercise training (sedentary) served as a control group. Similar to the human data, exposure of mice to the exercise protocol improved memory consolidation in the Morris water maze test, an established assay to test hippocampus-dependent spatial reference memory (Fig. 3B, C). While we could not detect reliable expression of microRNA-409-5p in the blood of mice, quantitative PCR (qPCR) analysis revealed that microRNA-501-3p was significantly increased after exercise (Fig. 3D). Moreover, both microRNAs were significantly increased in the hippocampus of exercising mice, when compared to the control group (Fig. 3C). In sum, these data support the view that microRNA-409-5p and microRNA-501-3p play an important role in the regulation of cognitive function and suggest that the mouse is a suitable model system for further analysis of the role of both microRNAs in the central nervous system.

### micoRNA-409-5p and 501-3p control structural synaptic plasticity

**Fig. 4:**
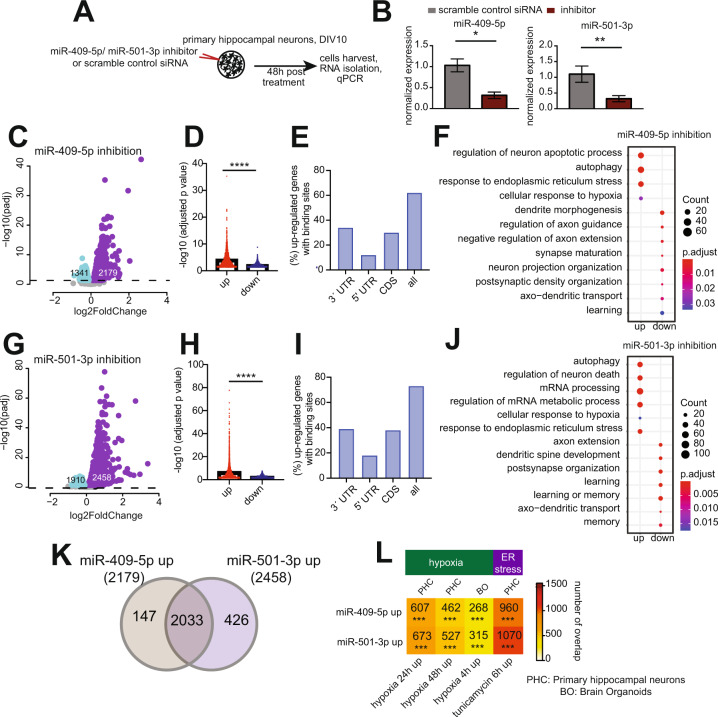
Inhibition of microRNA-409-5p and microRNA-501-3p affect gene-expression programs linked to neuronal integrity. **A** Experimental design. Primary hippocampal neurons were cultured for 10 days. Cells were then incubated with lipid nanoparticles (LNPs) containing anti-miRs or scramble control RNA for 48 h before RNA was isolated for sequencing. **B** Bar graphs showing the quantification of microRNA-409-5p and microRNA-501-3p expression by qPCR. *n* = 6/group, unpaired two-tailed *t* test. Error bar indicates mean ± SEM. **C** Volcano plot representing differentially expressed genes upon microRNA-409-5p knockdown. The *x*-axis displays fold changes (log2), blue represents downregulation, red represents upregulation of genes after anti-microRNA-409-5p treatment. The *y*-axis shows an adjusted *p* value (−log10). FDR < 0.05. **D** Barplot showing comparison of FDR adjusted *p* value distribution between up- and downregulated genes after silencing of microRNA-409-5p. ^****^p < 0.0001, Wilcoxon–Mann–Whitney test, two-tailed test, unpaired. Error bar = mean ± SEM. **E** Percentage of the up-regulated genes overlapped with the genes having predicted microRNA-409-5p binding sites at 3′ UTR, 5′ UTR, coding sequence (CDS) and all three combined (all). Genome-wide predicted microRNA binding sites were retrieved from miRWalk (version 3). **F** Gene ontology analysis of the deregulated genes. Dots on the left and represent up- and downregulated processes respectively. The size of the dots represents the number of genes belonging to each process. Color intensity represents adjusted *p* value. **G** Volcano plot representing differentially expressed genes upon microRNA-501-3p knockdown. **H** Barplot displays FDR adjusted *p* value distribution from up- and downregulated genes after microRNA-501-3p knockdown. ****p < 0.0001, Wilcoxon–Mann–Whitney test, two-tailed test, unpaired. Error bar = mean ± SEM. **I** Percentage of the microRNA-501-3p inhibition induced upregulated genes overlapped with the genes having predicted microRNA binding sites at 3′ UTR, 5′ UTR, coding sequence (CDS), and all three combined (all). Genome-wide predicted microRNA binding sites were retrieved from miRWalk (version 3). **J** Dot plot representing the deregulated gene ontology biological processes. **K** Overlap between up-regulated genes from microRNA-409-5p and microRNA-501-3p inhibition experiments. **L** Hypergeometric overlap of the deregulated genes to those from hypoxia and ER stress responses in neurons. Fisher’s exact test, adjusted *p* value with Benjamini Hochberg (BH) correction. Color map represents the number of overlapping genes. PHC represents primary hippocampal neurons, BO represents Brain organoids. PHC data were retrieved from [25] and BO data were retrieved from [54].

To further study the role microRNA-409-5p and microRNA-501-3p in neuronal plasticity we decided to inhibit their function in primary hippocampal neuronal cultures. LNPs packaged with inhibitors of microRNA-409-5p (anti-miR-409-5p) or microRNA-501-3p (anti-miR-501-3p) were applied to primary hippocampal neurons at DIV 10 and incubated for 48 h. Scramble oligonucleotides were used as control (Fig. 4A). Quantitative PCR results confirmed that the anti-miRs reduced the expression of their target microRNAs (Fig. 4B). With the aim to elucidate the cellular processes regulated by microRNA-409-5p and microRNA-501-3p we performed RNA-sequencing from primary neurons treated with either anti-miR-409-5p or anti-miR-501-3p. As expected, the inhibition of microRNA-409-5p or microRNA-501-3p led to up- and downregulation of many genes, while the up-regulation was more pronounced (Fig. 4C, D, G, H, Tables S4 and S5). A substantial amount of the upregulated genes possesses predicted microRNA binding sites (microRNA-409-5p: 62%; microRNA-501-3p: 73%) that are mainly located within their 3′untranslated (3′UTR) or coding regions (Fig. 4E, I). Gene ontology analyses revealed that the upregulated genes reflect processes such as the regulation of neuronal cell death, autophagy, hypoxia, and endoplasmic reticulum (ER) stress in the case of both microRNAs (Fig. 4F, J). Indeed, 93% of the genes upregulated after microRNA-409-5p inhibition overlap with those from microRNA-501-3p inhibition (Fig. 4K). We have recently analyzed the gene-expression changes in hippocampal neurons as a consequence of hypoxia and ER stress, which eventually leads to memory impairment [25]. Notably, the genes up-regulated in response to anti-miR-409-5p or anti-miR-501-3p treatment significantly overlapped with the genes increased as a consequence of hypoxia or ER-stress providing further support for the role of both microRNAs in the regulation of neuronal integrity (Fig. 4G). In line with these data, the genes downregulated in response to anti-miR-409-5p or anti-miR-501-3p treatment were linked to structural and functional synaptic plasticity (Fig. 4D, F). In sum, these data suggest that microRNA-409-5p and microRNA-501-3p are essential to maintain neuronal integrity and that loss of these microRNAs de-regulate cellular processes that will eventually lead to an impairment of synaptic plasticity, most likely as a secondary effect.

**Fig. 5:**
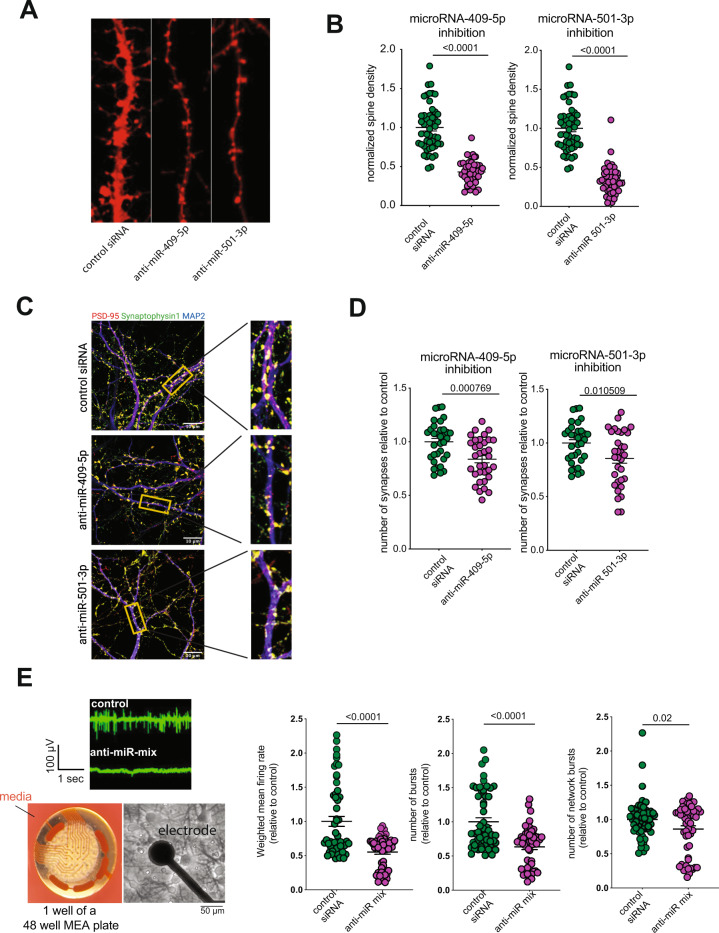
MicroRNA-409-5p and microRNA-501-3p control synaptic morphology and neuronal activity. **A** Representative confocal images of dendrites from scramble control and anti-miR treated neurons. **B** (Left panel) Spine density in anti-miR-409-5p and (Right panel) anti-miR-501-3p treated neurons in comparison to control. Dots represent dendritic segments acquired randomly from images. *y*-Axis shows the number of total spines per length of a chosen dendritic segment. (Left panel) number of dendritic segments analyzed, control siRNA = 49, anti-miR-409-5p = 41. (Right panel) number of dendritic segments analyzed, control siRNA = 49, anti-miR-501-3p = 48. Unpaired *t* test, two-tailed. Error bars = mean ± SEM. **C** Representative merged images of scramble control and anti-miRs treated neurons showing IHC staining for Synaptophysin 1, PSD-95, and MAP2. **D** Number of synapses in anti-miR-409-5p and anti-miR-501-3p treated neurons compared to those from scramble control-treated neurons. Dots represent individual images acquired randomly from the coverslips. Functional synapses were enumerated based on colocalized signals for PSD95 and Synaptophysin 1. *y*-Axis shows relative percentage of functional synapse in the treatment group(s) compared to controls. (Left panel) Number of images analyzed (control siRNA = 34, anti-miR-409-5p = 34). (Right panel) Number of images analyzed (control siRNA = 34, anti-miR-501-3p = 33). Unpaired *t* test, two-tailed. Error bar indicates mean ± SEM. **E** Primary hippocampal neurons were cultured on a Axion MEA plate and treated with lipid nanoparticles (LNPs) containing anti-miR cocktail of microRNA-409-5p and microRNA-501-3p at DIV 7. Neurons treated with scramble control were treated as controls. At DIV10, spontaneous neuronal activity was recorded at every 3 h for 10 min. The complete recording session lasted for 24 h. Left panel shows representative raw data traces from one electrode from a MEA plate from treatment and control groups (top) and representative low and high magnification images from a neuronal culture grown in one well of the MEA plate. The right panel shows from left to right thrree datasets; the Mean firing rate, the number of bursts and the number of network bursts after treatment with anti-miR-cocktail and scramble controls. *n* = 54/group, unpaired *t* test, two-tailed. Error bar indicates mean ± SEM.

These findings prompted us to analyze synaptic morphology in response to the down-regulation of microRNA-409-5p or microRNA-501-3p. Primary hippocampal neurons were treated with LNPs containing anti-miR-409-5p or anti-miR-501-3p. After 72 h of incubation, cells were investigated for cell viability and neurite outgrowth. Anti-miR-409-5p treatment significantly enhanced, while anti-miR-501-3p resulted in a non-significant trend for enhanced cell death. Neurite outgrowth was significantly impaired in anti-miR treated cells when compared to scramble control (Fig S4). Moreover, another batch of cells was fixed and stained for visualization of dendritic spines and mature synapses. As control, scramble small RNA containing LNPs were used. For the quantification of dendritic spines, we performed DiI staining (Fig. 5A). Quantitative analysis showed that neurons treated with either anti-miR-409-5p or anti-miR-501-3p displayed significantly reduced spine density when compared to scrambled control (Fig. 5B). To confirm this observation and more specifically analyze the number of mature synapses we performed immunohistochemical staining for the presynaptic marker protein Synaptophysin 1 and the postsynaptic marker protein postsynaptic density (PSD-95). In addition, neurons were stained for microtubule marker MAP2 to visualize dendrites (Fig. 5C). Colocalization of synaptophysin 1 and PSD-95 was used to quantify the number of mature synapses. The number of synapses was significantly reduced in neurons treated with either anti-miR-409-5p or anti-miR-501-3p when compared to the control group (Fig. 5D). These data show that both microRNAs are important for synaptic integrity and synapse number. Reduced synapse number is likely to affect neuronal activity. To test this, we analyzed neuronal cultures treated with a cocktail of anti-miR-409-5p and anti-miR-501-3p via a multielectrode array (MEA) assay. The preference for choosing the cocktail mixture over individual manipulation was based on the observation that both microRNAs share a large percentage of the downregulated genes (microRNA-409-5p: 85%, microRNA-501-3p: 60%) as well as the upregulated genes (microRNA-409-5p: 93%, microRNA-501-3p: 83%) when they were individually silenced (Fig. 4, Fig. S5) and their very similar effect on the neuronal integrity (Fig. 5A–D). To this end, primary hippocampal neurons were cultured in wells equipped with electrodes. At DIV 7 basal activity was first measured and then the cultures were treated with LNPs containing the mixture anti-miRs. After 72 h incubation the plate was transferred to MEA device for further recording. When compared to scramble controls, anti-miR-mixture treatment led to significant reduction in neuronal weighted mean firing rate, number of bursts and number of network bursts (Fig. 5E).

## Discussion

This work is in line with previous studies showing that exercise improves learning in rodents and humans [16, 26–29]. While we observed improved performance in the long-term verbal learning memory and WCST, it is important note that STM measured in the VLMT and the performance in the trial-making tests were not significantly improved upon exercise in our study. Thus, exercise-induced cognitive improvement likely affects different cognitive domains and would therefore be task and function specific. On the other hand, it is noteworthy that we observed an approaching trend for significant memory enhancement in all tests that did not reach significance. It is therefore also possible that differences in the dynamic range of the employed tests might play a role, especially since we analyzed healthy human subjects. This view is supported by previous studies showing that aerobic exercise improved short term memory function and performance in the trial-making tests when cognitively impaired individuals were analyzed, hence a ceiling effect could be excluded [22, 30, 31].Previous studies have analyzed circulating microRNAs in humans that underwent exercise but most of these studies aimed to correlate microRNA expression to other physiological parameters than cognitive function [32–38]. Using different integrative data analysis approaches we detected 5 microRNA clusters that were significantly altered upon exercise. However, only two of them showed a significant correlation to cognitive functions, with the midnightblue cluster being the most significant one that was therefore selected for further analysis. This observation also stresses the importance of our data analysis approach that not only aimed to identify microRNAs altered upon exercise but was designed to identify microRNAs potentially linked to changes in cognitive performance. Among the midnightblue cluster is microRNA-212 that has been linked to neuronal plasticity and memory function via rodent studies [24, 39, 40] thereby underscoring the validity of our approach. In this study, we decided to further study microRNA-409-5p and microRNA-501-3p as these microRNAs were amongst the most significantly regulated microRNAs of the midnightblue cluster and both were linked to exercise-improved cognitive function. Moreover, both microRNAs were highly expressed in the human and mouse brain. Importantly, similar to our human data, exercise also improved memory function in mice which was accompanied by the increased expression of microRNA-409-5p and microRNA-501-3p in the hippocampus. These data may suggest that exercise might also increase microRNA-409-5p and microRNA-501-3p in the human brain. At present we cannot conclusively answer the question if exercise-induced expression changes of microRNA-409-5p and microRNA-501-3p observed in blood and brain are functionally linked. It is, however, interesting to note that brain-derived microRNAs can reach the circulation via extracellular vesicles [10] and that microRNAs are known to mediate biological function across cells and organs [8, 41–43]. Thus, it is at least possible that brain-specific changes of microRNA expression may be observed in circulation. Clearly, more research is necessary to elucidate these processes. Taken together these findings hint to an important role of both microRNAs in neuronal functioning. Indeed, we found that the down-regulation of microRNA-409-5p and microRNA-501-3p in primary hippocampal neurons was associated with a reduced number of dendritic spines, mature synapses and reduced neuronal network activity. Moreover, knockdown of microRNA-409-5p or microRNA-501-3p in hippocampal neurons induced a dysregulation of gene expression. On one hand, genes linked to cellular processes such as autophagy, cell death, hypoxia, and ER stress were massively upregulated. The de-regulation of these processes is known to have a negative impact on neuronal function [25, 44–47]. Indeed, genes linked to spine development, synapse maturation, learning and memory were down-regulated. These findings suggest that reduced microRNA-409-5p and microRNA-501-3p levels might first induce destructive/deleterious cellular processes, which might then impair synaptic function. One could speculate that this could in turn weaken learning and memory abilities and may promote neuropsychiatric and neurodegenerative disease. In turn, higher levels of microRNA-409-5p and microRNA-501-3p would be associated with improved neuronal function and learning abilities. The role of microRNA-501-3p and microRNA-409-5p in human cognition is also supported by the literature. For example, microRNA-501-3p was found to be downregulated in serum of patients with Alzheimer’s disease (AD) and its levels were also correlated with the cognitive score [48]. In line with these data, a recent study identified plasma levels of microRNA-501-3p as a predictor of cognitive performance in elderly individuals [49]. No data from humans is available for microRNA-409-5p but expression changes have been observed in rodent models for neurodegenerative diseases. For example, microRNA-409-5p was significantly downregulated when whole-brain tissue from a mouse model of AD was compared to control littermates [50]. Similarly, decreased microRNA-409-5p expression was observed in the brain of mice that were subjected to an experimental model of isoflurane-induced cognitive impairment [51]. The regulation of microRNA-501-3p and microRNA-409-5p in AD and upon exercise is thus also in agreement with experimental and epidemiological data suggesting that physical exercise can help to prevent and ameliorate cognitive decline in AD [52, 53]. Future studies will aim to identify the precise mechanisms and immediate target mRNAs by which microRNA-409-5p and microRNA-501-3p affect neuronal plasticity. Moreover, while we focused on microRNA-409-5p and microRNA-501-3p in this study, it will be interesting to analyze the role of the other microRNAs of the midnightblue but also the MEtan cluster in more detail.

In conclusion, our data suggest that exercise-mediated cognitive enhancement in healthy humans provides an important approach to identify microRNAs potentially linked to cognitive function in humans. The cross-correlation of such data with the available data from disease models and patients and the combination with subsequent mechanistic studies in animal models will help to identify key microRNAs that may serve as biomarkers or drug targets to treat cognitive diseases.

## Supplementary information

Supplemental figure legends

Supplemental Figure 1

Supplemental Figure 2

Supplemental Figure 3

Supplemental Figure 4

Supplemental Figure 5

Supplemental table 1

Supplemental table 2

Supplemental table 3

Supplemental table 4

Supplemental table 5

## Data Availability

De-identified and processed sequencing data related to humans are available from the corresponding author upon reasonable request. Sequencing data related to mouse experiments are available at NCBI GEO (accession ID: GSE17163). The data analysis has been performed in R (v 4.0.5) using freely available packages. All packages are available from CRAN or Bioconductor.
