# Supplementary material for: Exercise as a model to identify microRNAs linked to human cognition: a role for microRNA-409 and microRNA-501
Source: Transl Psychiatry. 2021 Oct 8;11:514. doi: 10.1038/s41398-021-01627-w (PMC8501071; doi:10.1038/s41398-021-01627-w)
Supplement: Supplementary file 1 — Supplemental figure legends [file 41398_2021_1627_MOESM1_ESM.docx]

**Supplemental figure legends**

**Fig S1: Exercise as a model to identify microRNAs linked to human cognition. A.** Experimental Scheme. **T**o identify microRNA potentially linked to human cognition, we employed an aerobic exercise model, since exercise is known to improve cognitive function in healthy humans. Thus, healthy subjects were subjected to exercise and blood samples collected pre- and post-exercise were used to perform smallRNA-sequencing. **B.** Via an integrative data analysis approach we aimed to identify microRNA correlated to cognitive function. **C.** As a final step we planned to test candidate microRNA for their role in structural and functional synaptic plasticity.

**Fig S2: Effects of exercise in healthy humans. A.** Experimental setting. Healthy humans (n =19, 14 male, 5 female) were subjected to a 3-month exercise training protocol. **B.** Body weight was similar between pre- and post-exercise measurements. **C.** Cardiac parameters such as pulse, systolic **(D)** and diastolic **(E)** blood pressure were not significantly altered when comparing individuals pre- vs. post-exercise. There was a trend for reduced systolic blood pressure post-exercise. **F.** Blood levels of triglyceride, cholesterol **(G)** and glucose level **(H)** were not significantly altered upon exercise. **I.** Short term memory (STM) measured via the VLMT was not significantly altered post-exercise, although there was a non-significant trend towards increased performance. **J.** Performance in the TMT-A and the TMT-B **(K)** was not significantly altered post-exercise. A non-significant trend for better performance was noted in both versions of the TMT. n=19/group, the horizontal line in the box plot represents median, the box spans 25% and 75% quantile, and the whiskers represent the smallest and largest values in the 1.5x interquartile range.

**Fig S3: Weighted microRNA co-expression analysis and correlation with clinical traits and cognitive performance.** WGNCA analysis identified 33 microRNA co-expression modules of which 28 modules are shown here. The other 5 modules are depicted in Fig 1. Each row represents an expression module (ME) while each column corresponds to a phenotypic trait. Each cell shows the corresponding correlation. The p-values are shown in parentheses. The values are color-coded based on direction and degree of correlation, blue represents negative correlation while red represents positive correlation.

**Fig S4: Effect of anti-miR-409-5p and anti-miR-501-3p on neuronal viability and neurite outgrowth.** Primary hippocampal neurons were cultured and treated with lipid nanoparticles (LNPs) containing anti-miR cocktail of microRNA-409-5p and microRNA-501-3p at DIV 7. Neurons treated with scramble control were treated as controls. At DIV10 neurons were examined for cell viability and neurite outgrowth using ReadyProbes® Cell Viability and Neurite Outgrowth Staining kits respectively (see methods for details). **A.** NucGreen/Nucblue signal (dead/all cells) ratio in anti-miR-409-5p and anti-miR-501-3p treated neurons. Signals are normalized to those from neurons treated with scramble control. **B.** Comparison of neurite outgrowth fluorescence signal between anti-miR and scramble control treated neurons. Fluorescence signal in treatment group is normalized to that from control group. Unpaired t-test, n=5/group.

**Fig S5: Comparison between anti-miR-409-5p and anti-miR-501-3p induced deregulated genes.** Venn diagram showing the high overlap between the downregulated genes from experiments related to microRNA-409-5p and microRNA-501-3p inhibition in primary hippocampal neurons.
