# Supplementary material for: Exercise as a model to identify microRNAs linked to human cognition: a role for microRNA-409 and microRNA-501
Source: Transl Psychiatry. 2021 Oct 8;11:514. doi: 10.1038/s41398-021-01627-w (PMC8501071; doi:10.1038/s41398-021-01627-w)
Supplement: Supplementary file 2 — Supplemental Figure 1 [file 41398_2021_1627_MOESM2_ESM.pdf]

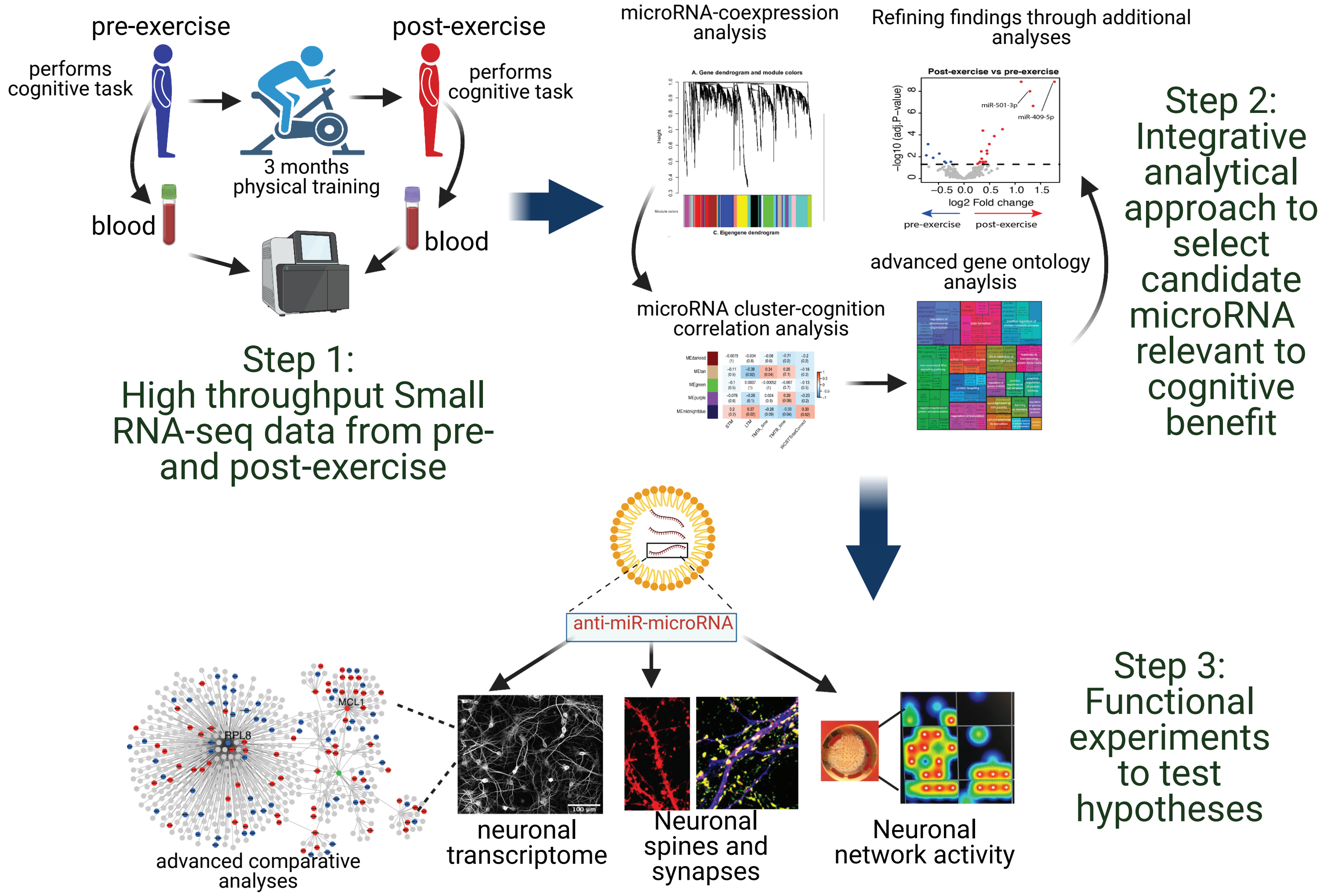

pre-exercise
performs cognitive task
post-exercise
performs cognitive task
3 months physical training
blood
blood
Step 1: High throughput Small RNA-seq data from pre- and post-exercise
microRNA-coexpression analysis
Refining findings through additional analyses
Post-exercise vs pre-exercise
miR-501-3p
miR-409-5p
log2 Fold change
pre-exercise
post-exercise
advanced gene ontology anaylsis
microRNA cluster-cognition correlation analysis
Step 2: Integrative analytical approach to select candidate microRNA relevant to cognitive benefit
anti-miR-microRNA
RPL8
MCL1
advanced comparative analyses
neuronal transcriptome
Neuronal spines and synapses
Neuronal network activity
Step 3: Functional experiments to test hypotheses
