# Supplementary figures and images for: Exercise as a model to identify microRNAs linked to human cognition: a role for microRNA-409 and microRNA-501
Source: Transl Psychiatry. 2021 Oct 8;11:514. doi: 10.1038/s41398-021-01627-w (PMC8501071; doi:10.1038/s41398-021-01627-w)

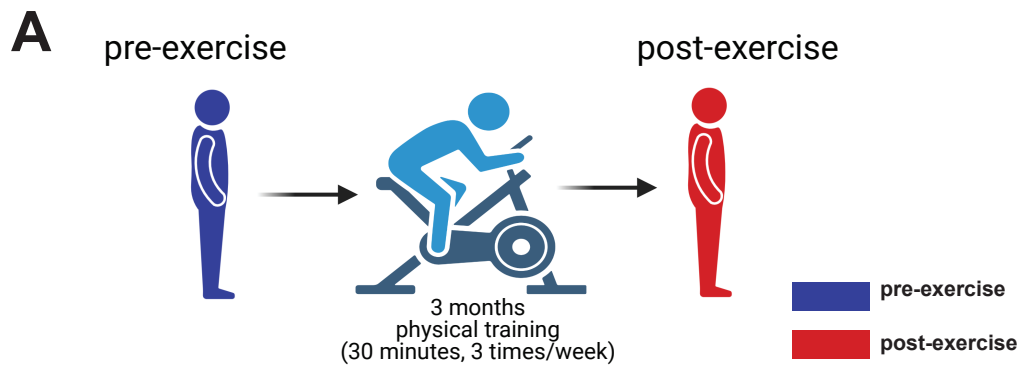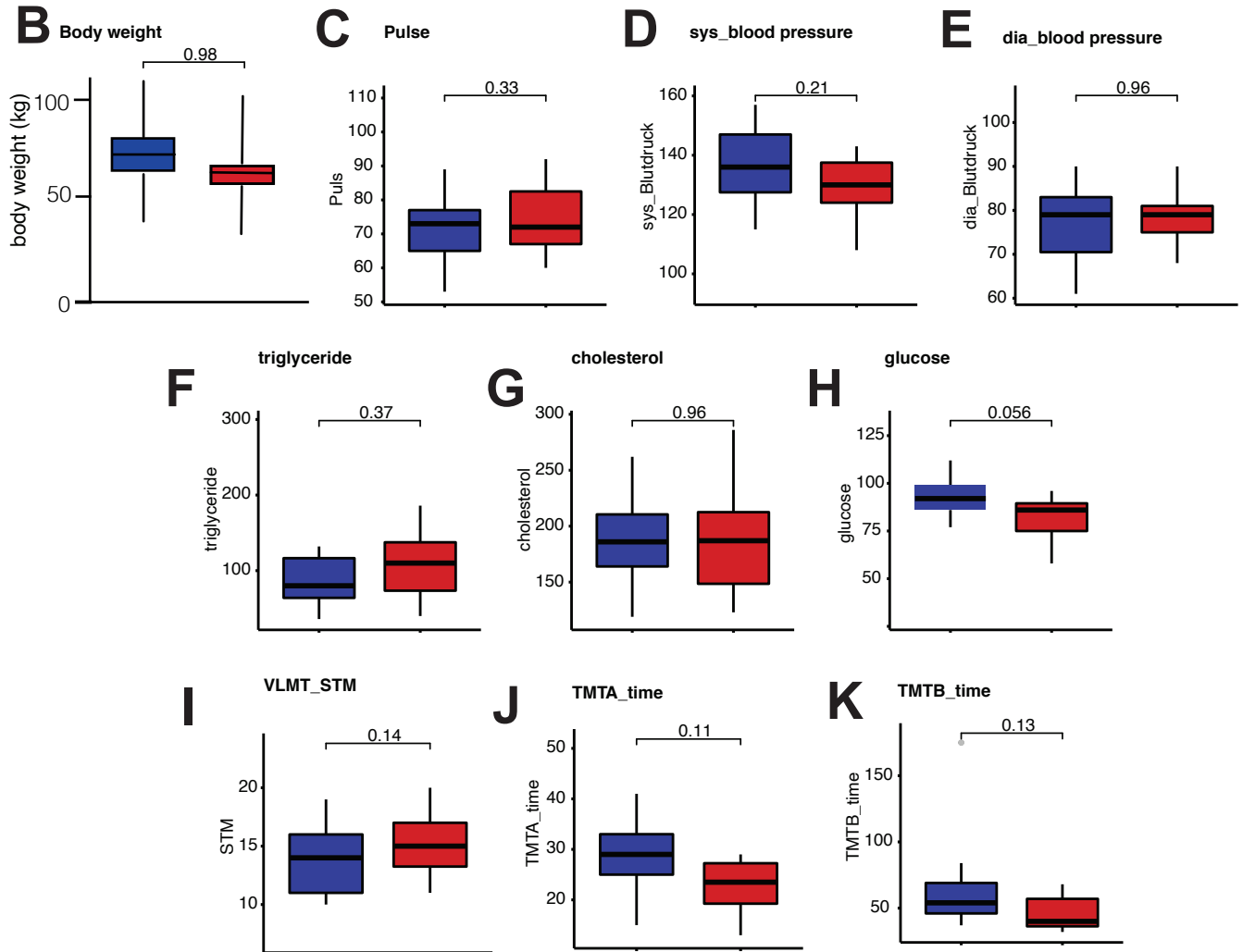

Supplement: Supplementary file 3 — Supplemental Figure 2 [file 41398_2021_1627_MOESM3_ESM.pdf]

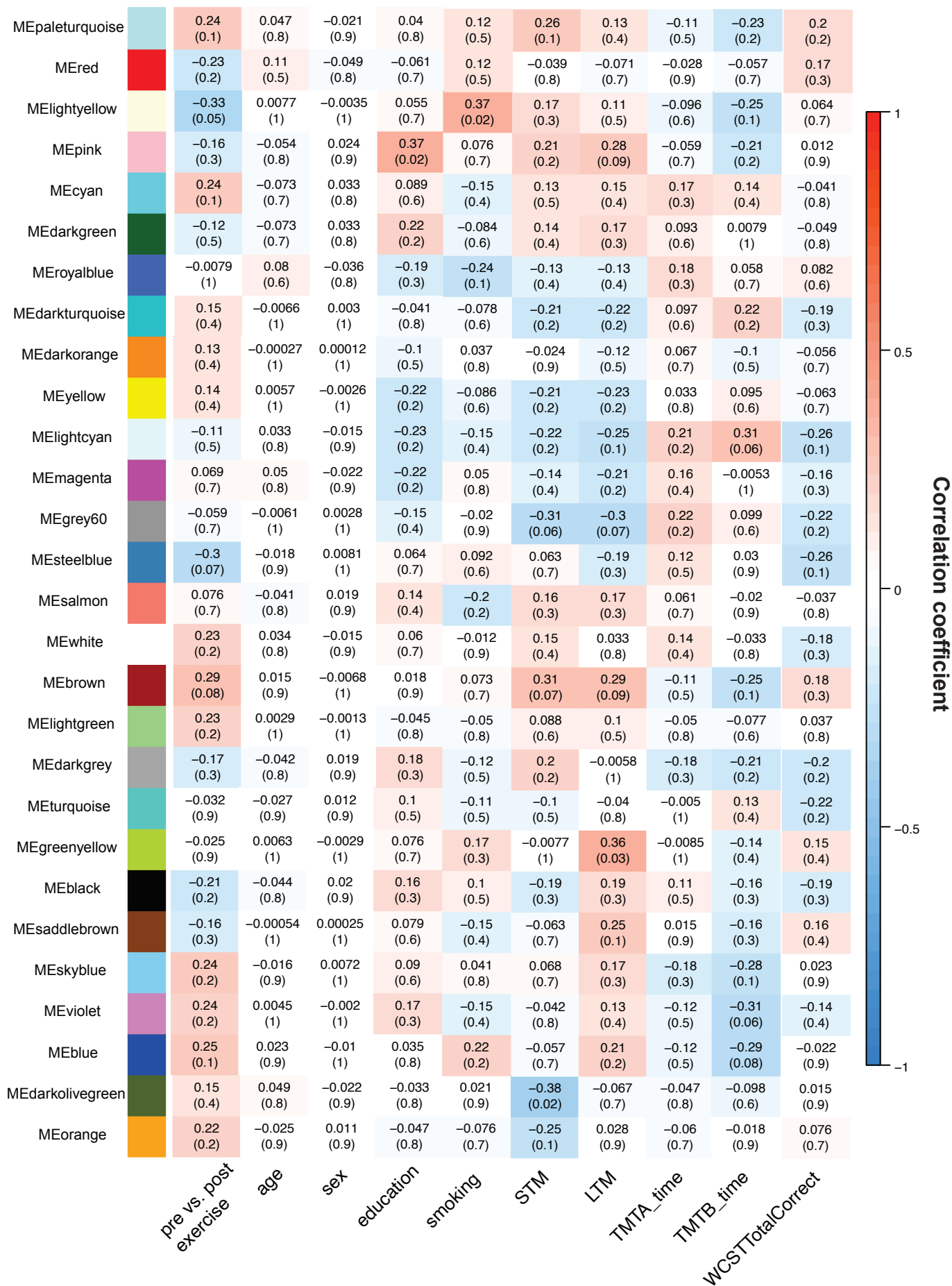

Supplement: Supplementary file 4 — Supplemental Figure 3 [file 41398_2021_1627_MOESM4_ESM.pdf]

A

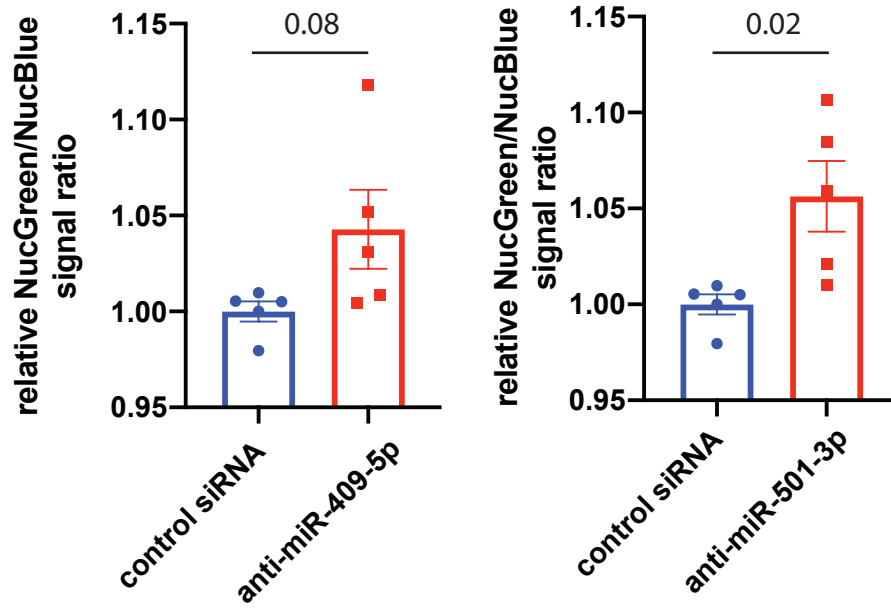

B

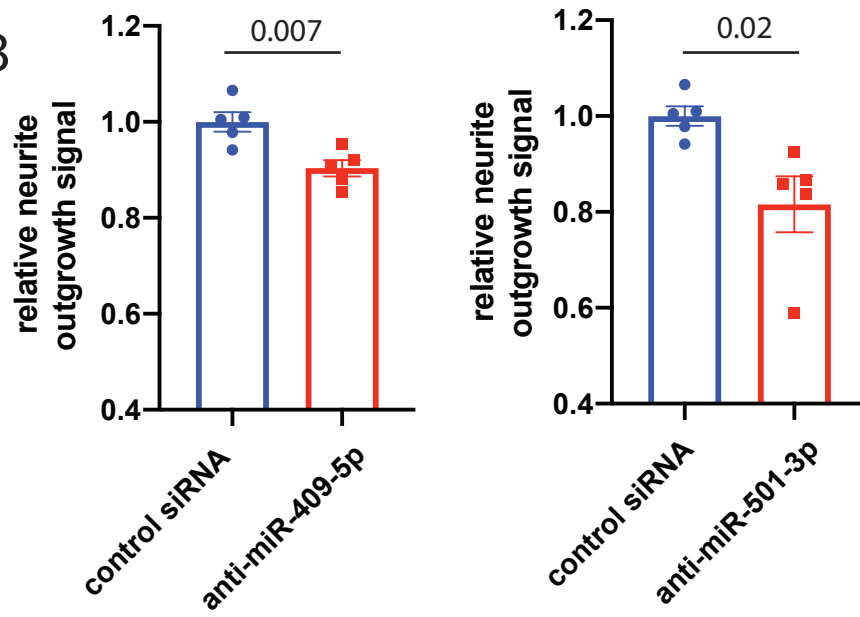

Supplement: Supplementary file 5 — Supplemental Figure 4 [file 41398_2021_1627_MOESM5_ESM.pdf]

miR-409-5p down  
(1341)

miR-501-3p down  
(1911)

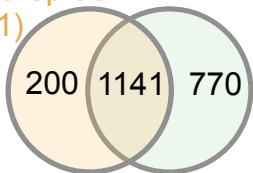

Supplement: Supplementary file 6 — Supplemental Figure 5 [file 41398_2021_1627_MOESM6_ESM.pdf]
